# Orangutan Alu quiescence reveals possible source element: support for ancient backseat drivers

**DOI:** 10.1186/1759-8753-3-8

**Published:** 2012-04-30

**Authors:** Jerilyn A Walker, Miriam K Konkel, Brygg Ullmer, Christopher P Monceaux, Oliver A Ryder, Robert Hubley, Arian FA Smit, Mark A Batzer

**Affiliations:** 1 Department of Biological Sciences, Louisiana State University, 202 Life Sciences Building, Baton Rouge, LA 70803, USA; 2 Department of Computer Science, Center for Computation and Technology (CCT), Louisiana State University, 316 Johnston Hall, Baton Rouge, LA 70803, USA; 3 Department of Molecular and Cellular Physiology, Louisiana State University Health Sciences Center, 1501 Kings Highway, Shreveport, LA 71130, USA; 4 School of Biological Sciences, Louisiana Tech University, Ruston, LA 71272, USA; 5 Conservation and Research for Endangered Species (CRES), Zoological Society of San Diego, San Diego, CA 92112, USA; 6 Institute for Systems Biology, Seattle, WA 98103, USA

## Abstract

**Background:**

Sequence analysis of the orangutan genome revealed that recent proliferative activity of *Alu *elements has been uncharacteristically quiescent in the *Pongo *(orangutan) lineage, compared with all previously studied primate genomes. With relatively few young polymorphic insertions, the genomic landscape of the orangutan seemed like the ideal place to search for a driver, or source element, of *Alu *retrotransposition.

**Results:**

Here we report the identification of a nearly pristine insertion possessing all the known putative hallmarks of a retrotranspositionally competent *Alu *element. It is located in an intronic sequence of the *DGKB *gene on chromosome 7 and is highly conserved in *Hominidae *(the great apes), but absent from *Hylobatidae *(gibbon and siamang). We provide evidence for the evolution of a lineage-specific subfamily of this shared *Alu *insertion in orangutans and possibly the lineage leading to humans. In the orangutan genome, this insertion contains three orangutan-specific diagnostic mutations which are characteristic of the youngest polymorphic *Alu *subfamily, *Alu*Ye5b5_*Pongo*. In the *Homininae *lineage (human, chimpanzee and gorilla), this insertion has acquired three different mutations which are also found in a single human-specific *Alu *insertion.

**Conclusions:**

This seemingly stealth-like amplification, ongoing at a very low rate over millions of years of evolution, suggests that this shared insertion may represent an ancient backseat driver of *Alu *element expansion.

## Background

The amplification of *Alu *elements has been ongoing in primate genomes for about 65 million years [1,2]. They typically mobilize via a 'copy and paste' mechanism through an RNA intermediate, a process termed target-primed reverse transcription (TPRT) [3]. *Alu *elements are non-autonomous and utilize the enzymatic machinery of autonomous LINE elements (L1) to mobilize [1,4,5]. Due to the staggered DNA cuts of the genome by the L1-derived endonuclease during TPRT, *Alu *insertions are flanked by short sequences of duplicated host DNA called target site duplications (TSDs), which can be used to identify the insertion event. *Alu *elements accumulate in an 'identical by descent' manner. This means that the ancestral state at any locus is the absence of the element and, conversely, that the presence of a shared element with matching TSDs at a given locus indicates a common ancestor. Thus, *Alu *elements are considered essentially homoplasy-free characters [1,6]. Although the autonomous features of L1 are straightforward, the identification of *Alu *element insertions that retain the ability to propagate copies of themselves has remained somewhat elusive. This is primarily because *Alu *elements do not contain coding sequence and the vast majority of insertions are highly similar to each other. Structural factors, such as having an intact promoter region, low sequence diversity from a known polymorphic subfamily, close proximity of the polymerase III (Pol III) termination signal to the end of the element and the length of the poly(A) tail, have all been associated with *Alu *retrotransposition ability [4,7,8]. Yet, to date, only one *Alu *source element has been identified in humans with clear evidence that it produced an offspring element [4]. This rare finding is due in part to the large landscape of hundreds of relatively young elements with limited knowledge about what characteristics make them retrotransposition competent. In the case of the orangutan, the landscape of relatively young elements is quite sparse [9].

Orangutans are characterized by a relatively long lifespan among primates (35 to 45 years in the wild) combined with the longest average inter-birth interval between offspring (8 years) [9,10]. These relatively low reproduction rates along with their relatively large body size compared to other great apes are consistent with a 'slow' life history strategy, impacting their genomic architecture over time. Investigation of the orangutan draft genome sequence [ponAbe2] revealed a very low retrotransposition rate of *Alu *elements in the orangutan lineage leading to *Pongo abelii *(Sumatran orangutan), while seeming to maintain an L1 activity comparable to other primates [9]. This finding was in particular startling because all primate genomes studied to date showed evidence of strong ongoing *Alu *and L1 retrotransposition [11-13]. Variation in *Alu *retrotransposition activity within different primate species has been reported previously [14] and is known to vary over the course of evolution [15], but the orangutan genome provided the first evidence of such a dramatic decline in *Alu *retrotransposition in primates. An extensive analysis of the ponAbe2 assembly identified only approximately 250 lineage-specific *Alu *insertions, which translates to an average of only about 18 new insertions per million years [9]. This is in sharp contrast to analyses of the human and chimpanzee genomes, in which approximately 5,000 and 2,300 lineage-specific *Alu *insertions were identified, respectively [9,11,13]. Of the orangutan-specific *Alu *subfamilies, three were determined to be the youngest. We have termed these *Alu*Yc1a5_*Pongo, Alu*Ye5a2_*Pongo *and *Alu*Ye5b5_*Pongo *based on sequence comparisons to previously identified human *Alu *subfamilies and using the standardized nomenclature for *Alu *repeats [16] (see methods for naming convention). From these three youngest orangutan *Alu *subfamilies, the 44 youngest appearing elements, on the basis of divergence from their subfamily consensus sequences, were analyzed for insertion presence or absence in a DNA panel of orangutans and other primates for the population genetics portion of the Orangutan Genome Project [9] (supplementary section 19); and only 13 were shown to be polymorphic in the orangutans evaluated. We postulated that the low number of recent *Alu *insertions representing only three young subfamilies might provide an ideal genomic landscape to search for their source elements.

## Results

We used BLAT [17] to computationally search the orangutan ponAbe2 genome assembly for the matches with the highest homology to each of the three polymorphic subfamily consensus sequences. The vast majority of the closest matches were young appearing (low divergence from their respective consensus sequences) loci already evaluated by PCR in the previous study [9]. However, the results for the closest matches to the *Alu*Ye5b5_*Pongo *consensus sequence revealed a previously uncharacterized *Alu *insertion on chromosome 7 (Chr7) with > 99% sequence identity to the consensus sequence (Table 1). Using the University of California Santa Cruz (UCSC) Genome Browser [18,19], it became evident that this insertion was not a recent event overlooked by our previous analyses, but was shared by the human (hg18) and chimpanzee (panTro2) genomes, while absent from the rhesus macaque genome (rheMac2). Therefore, this locus had been computationally filtered from our previous study of young orangutan-specific *Alu *insertions because it was also present in the human genome.

**Table 1:** Orangutan BLAT [ponAbe2] results for *Alu *subfamily *Alu*Ye5b5_*Pongo*

Identity (%)	Chromosome	Start	End	Distribution	Frequency
99.70	12	90005006	90005290	Poly Sum/Bornean	0.5405

99.30	7	70375547	70375831	Shared H/C/G/O	1.0000

98.60	17	56932716	56932999	Poly Sum specific	0.0811

99.30	13	109637257	109637541	Poly Sum specific	0.2027

98.30	21	23655335	23655618	Poly Sum specific	0.0946

97.90%	2b	3809376	3809659	Poly Sum specific	0.3513

H/C/G/O: human, chimpanzee, gorilla, orangutan; Poly: polymorphic; Sum: Sumatran.

**Figure 1:**
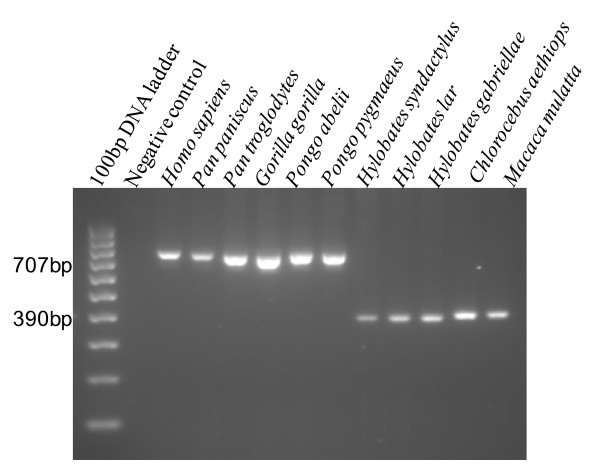
**Analysis of the chromosome 7 shared *Alu *insertion using polymerase chain reaction**. The *Alu *insertion is shared among *Hominidae *(the great apes) and absent from other extant primates. The filled site is approximately 707 bp (lanes 3 to 8) and the empty site is 390 bp (lanes 9 to 13). Lanes: (1) 100 bp DNA ladder; (2) negative control; (3) human; (4) bonobo chimpanzee; (5) common chimpanzee; (6) lowland gorilla; (7) Sumatran orangutan; (8) Bornean orangutan; (9) siamang; (10) white-handed gibbon; (11) red-cheeked gibbon; (12) African green monkey; (13) rhesus macaque.

The Genome Browser function [18,19] indicated that this shared Chr7 *Alu *insertion is located in an intronic sequence of the *DGKB *gene (coding for diacylglycerol kinase beta enzyme) [20]. The *DGKB *gene is one of several mammalian genes that encode for diacylglycerol kinases. Diacylglycerol kinases are involved in cellular processes by regulating diacylglycerol levels. For *DGKB*, two alternatively spliced transcript variants have been identified [21]. PCR evidence confirmed the presence of this Chr7 *Alu *insertion in human, chimpanzee, gorilla and orangutan, and indicated the absence from siamang, gibbon, African green monkey and rhesus macaque (Figure 1), suggesting this element is specific to *Hominidae *(the great apes) and absent from *Hylobatidae *(the gibbons and siamang) and other extant primates. This dates the insertion event to at least 16 million years ago using species divergence estimates from Locke *et al. *[9].

**Figure 2:**
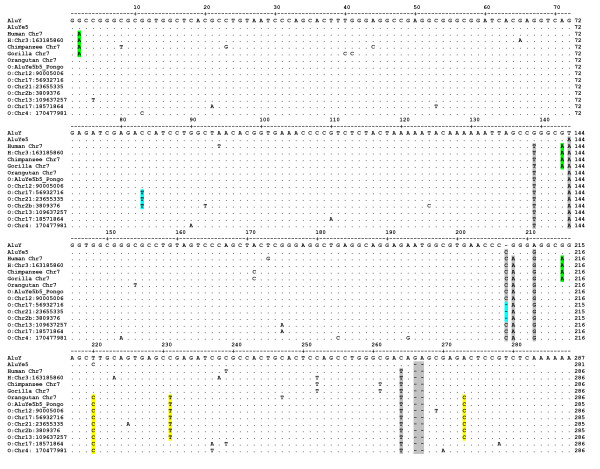
***Alu *Sequence alignment**. The consensus sequence for the ancestral *Alu*Y subfamily is shown at the top. The dots represent the same nucleotide as *Alu*Y. Deletions are shown as dashes and mutations are shown as the corrected base. The chromosome 7 locus has a number of mutations different from *Alu*Y that are shared by all investigated species (highlighted in gray) and are all located in the right monomer of the element following the middle A-rich region. Post-insertion, the chromosome 7 locus in the orangutan (labeled Orangutan Chr7) independently acquired sequential diagnostic mutations (highlighted in yellow) shared by all polymorphic loci of the young *Alu*Ye5b5_*Pongo *subfamily in orangutans (starting with O:Chr). At some point, one of the *Alu*Ye5b5_*Pongo *members subsequently acquired one substitution and one deletion (highlighted in aqua) and has propagated as a daughter subfamily. Following the divergence of orangutan and the lineage leading to humans, the chromosome 7 locus acquired three substitutions (highlighted in green) shared in gorilla, chimpanzee and human. There is one human-specific *Alu *insertion, H: Chr3, which shares these three variants.

An alignment of the BLAT sequence from human (hg18), chimpanzee (panTro2) and orangutan (ponAbe2) revealed a remarkably conserved left monomer and few nucleotide substitutions in the right monomer in all three species (Figure 2 and Additional file 1: Figure S1). In order to verify the PCR data of a shared insertion, DNA from eight different orangutans (four Sumatran and four Bornean), a lowland gorilla and two pygmy chimpanzees (bonobo) were PCR amplified at this locus. PCR products were gel purified, cloned and Sanger sequenced to confirm that the homologous insertions shared matching TSDs and flanking sequence. A sequence alignment of these results is shown in Additional file 1: Figure S1 and a list of the DNA samples used for sequencing is shown in Additional file 1: Table S1. The left monomer of the *Alu *element is highly conserved, especially among the orangutans, and all species share the TTTT Pol III transcription termination signal in the 3' TSD. These sequencing results confirmed that the Chr7 *Alu *element was shared among all great ape species, that the sequence in all individual orangutans was nearly identical to the *Alu*Ye5b5_*Pongo *consensus sequence and that it possessed some traditional hallmarks associated with retro transposition ability.

Members of the orangutan *Alu*Ye5b5_*Pongo *subfamily most closely matching the Chr7 *Alu *consensus sequence from the ponAbe2 genome assembly (n = 7 loci) were evaluated by PCR for presence or absence and allele frequency distribution (Table 2) in a DNA panel including 37 orangutans (see Methods and Additional file 2: Table S2; Genotypes/DNA samples). Next, we computationally searched the chimpanzee genome (panTro2) and human genome (hg18) for the closest matches to their respective Chr7 *Alu *consensus sequences using BLAT. BLAT results for the human genome identified one *Alu *insertion on chromosome 3 that was > 97% identical (Table 3) and shared the same three substitutions, suggesting that it may have derived from the Chr7 source locus. PCR primers were designed for the chromosome 3 locus and genotyped on a population panel of 80 human DNA samples representing four world populations (Additional file 2: Table S2; DNA samples). The chromosome 3 insertion was determined to be human specific but fixed present in all individuals tested. A sequence alignment of these results for orangutan and human is shown in Figure 2. We could not identify any candidate loci in the chimpanzee genome. The closest match was only about 95% identical and did not share any of the *Pan *lineage-specific point mutations of the panTro2 Chr7 insertion (see Additional file 1: Figure S1).

**Table 2:** Orangutan BLAT [ponAbe2] results for orangutan chromosome 7 locus

Identity (%)	Chromosome	Start	End	Distribution	Frequency
100.00	7	70375547	70375836	Shared H/C/G/O	1.0000

99.00	12	90005006	90005295	Poly Sum/Bornean	0.5405

98.00	17	56932716	56933004	Poly Sum specific	0.0811

98.70	13	109637257	109637546	Poly Sum specific	0.2027

97.60	21	23655335	23655623	Poly Sum specific	0.0946

97.30	2b	3809376	3809664	Poly Sum specific	0.3513

96.30	17	18571864	18572153	Orangutan specific	fixed

95.90	4	170477976	170478265	Orangutan specific	fixed

H/C/G/O: human, chimpanzee, gorilla, orangutan; Poly: polymorphic; Sum: Sumatran.

**Table 3:** Human BLAT [hg18] results for human chromosome 7 locus

Identity (%)	Chromosome	Start	End	Distribution	Frequency
100.00	7	14399134	14399438	Shared H/C/G/O	1.0000

97.10	3	163185859	163186163	Human specific	fixed

H/C/G/O: human, chimpanzee, gorilla, orangutan.

Figure 2 shows that the Chr7 locus has a number of mutations different from the ancestral *Alu*Y consensus sequence that are shared in all the great ape species. These are highlighted in gray. Of the previously characterized *Alu *subfamilies, the Chr7 locus most closely matches the *Alu*Ye5 subfamily [22,23]. From this we can infer that the Chr7 locus was a member of the *Alu*Ye lineage upon its insertion. Figure 2 further illustrates that, post-insertion, lineage-specific point mutations have occurred at this locus in the various species over time. In the orangutan, the Chr7 *Alu *(hereafter designated as O:Chr7) left monomer has remained completely unscathed by about 16 million years of evolution and has no mutations compared to the ancestral *Alu*Y consensus sequence. The O:Chr7 right monomer independently acquired three sequential nucleotide substitutions (highlighted in yellow) which are also diagnostic mutations that define the young polymorphic *Alu*Ye5b5_*Pongo *subfamily. The first, at position 220, coincides with one of the diagnostic variants characteristic of the *Alu*Ye5 subfamily. Therefore it is possible that this is not a post-insertion orangutan-specific substitution, but rather was present upon insertion and later experienced a backward mutation in the common ancestor of gorilla, chimpanzee and human. Regardless, the other two of these three diagnostic substitutions in O:Chr7 are also present in all five of the youngest polymorphic *Alu*Ye5b5_*Pongo *elements in orangutan and are absent from the two fixed insertions. In gorilla, chimpanzee and human, the Chr7 insertion has acquired three different shared mutations (highlighted in green). This alignment (Figure 2) provides strong evidence for the evolution of lineage-specific *Alu *insertion events from the ancestral Chr7 source element. There is further evidence that at least one of the orangutan-specific insertions, after acquiring two additional mutations (highlighted in aqua), remained active as a secondary source element generating new daughter copies.

**Figure 3:**
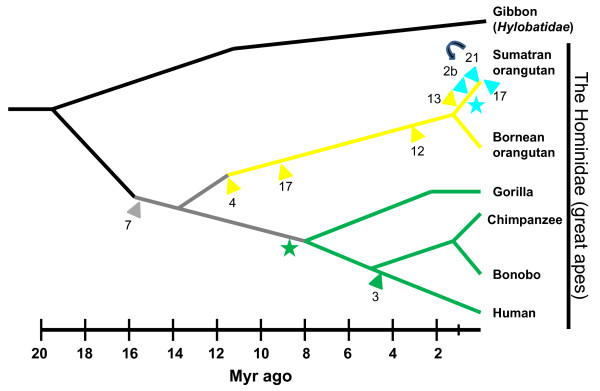
**A schematic of predicted *Alu *insertion events illustrated on a primate evolutionary tree from gibbon (a small ape) to the great apes**. The estimated evolutionary time period is shown on the scale at the bottom in millions of years (Myr). The gray arrowhead depicts the basal *Alu *insertion event on chromosome 7 shared among the great apes. The yellow arrowheads represent the approximate time period, based on nucleotide divergence and allele frequencies of offspring *Alu *insertion events specific to the orangutan lineage. The aqua star represents the emergence of a daughter *Alu *subfamily, based on diagnostic mutations, and the aqua arrowheads correspond to subsequent offspring *Alu *insertion events. The green star represents the approximate time period when the chromosome 7 parent element acquired two additional CpG and one non-CpG nucleotide substitutions shared by human, chimpanzee and gorilla (green lines). The green arrowhead denotes a subsequent human-specific *Alu *insertion event sharing these three variants. The numbers at the base of the arrowheads indicate the chromosomal locations. The curved arrow between insertions on 2b and 21 (Sumatran-specific polymorphic *Alu *insertions) indicates an alternative order of insertion.

These data provide strong evidence that the Chr7 ancestral *Alu *insertion underwent a hierarchical accumulation of multiple post-insertion diagnostic substitutions in the orangutan, while also failing to accumulate the more likely random variants over the same evolutionary time period. This provides strong support that the Chr7 *Alu *insertion is the source element, or the original founder element, from which the younger, lineage-specific *Alu *insertions shown in Figure 2 derived. By counting the number of non-diagnostic random mutations that have occurred in each of the proposed offspring elements in the orangutan (accounting for CpG versus non-CpG) and using the calculated allele frequency distribution for each locus as determined by PCR (Tables 1 and 2 and Additional file 3: Table S2; genotypes and allele frequencies), we constructed an estimated time-scale schematic for the propagation of this *Alu *lineage from the founder Chr7 locus (Figure 3). The evolutionary order in which each lineage-specific *Alu *insertion occurred is relatively straightforward along the majority of the estimated time-scale. However, it is not precisely clear which element represents the secondary source *Alu *that sprouted the most recent Sumatran orangutan-specific subfamily shown in aqua. The O:Chr2b locus has the most non-CpG random mutations and the highest allele frequency (0.3513), indicating it is likely the oldest of the three insertions (Additional file 3: Table S2). However, this element integrated into the middle of an existing L1 element (Additional file 3) and presumably is unable to retrotranspose from this location. It is also exceedingly unlikely that all three loci accumulated the same two mutations independently post-insertion. Rather, it is much more likely that either one gave rise to the other two or, alternatively, the source of all three loci is no longer evident in the orangutan genome. Given that the O:Chr17 locus appears the most recent with a very low allele frequency (0.0811), then the O:Chr21 locus appears most likely to be the secondary source driver if we assume that one necessarily gave rise to the other two. However, it seems more plausible that an unidentified secondary source driver produced all three copies and is simply not present in the ponAbe2 assembly or has been lost from the genome due to lineage sorting. These alternative scenarios are depicted at the terminal branches of Figure 3.

## Discussion

It is widely accepted that the expansion of *Alu *elements in primate genomes has occurred by using the L1 element enzymatic machinery for retrotransposition [1,5]. The identification of retrotranspositionally competent L1 elements is relatively straightforward as only full-length elements having both open reading frames completely intact are capable of propagation via TPRT [24]. Only a limited number of L1 elements meet these criteria as the vast majority of L1s in primate genomes are truncated or have other disabling mutations [25]. The identification of potentially active *Alu *source elements is far more complicated because the majority of *Alu *elements are full-length and they do not contain a coding sequence. Recent research has investigated several structural features that influence the ability of *Alu *elements to replicate. These include the upstream flanking sequence, the integrity of the left monomer, the sequence identity to a known polymorphic subfamily, the distance of the Pol III termination signal from the 3' end of the element and the length and integrity of the poly(A) tail. A discussion of these factors supports the candidacy of our Chr7 *Alu *insertion as an ancestral source element.

The upstream flanking sequence of an *Alu *element has been reported to influence transcription ability [26-28]. The Chr7 *Alu *element reported here has what appears to be an intact TATA box (5'TATAAAAA3') cis regulatory transcription promoter immediately upstream to the 5' TSD that is conserved in all species (Additional file 1: Figure S1). Although a TATA box is typically about 25 bp upstream of a transcription site and is usually the binding site for RNA polymerase II [29], TATA-box-like promoter sequences have been linked to the efficient transcription of the *Alu*-like human 7SL RNA gene by RNA polymerase III *in vitro *[30]. In addition, the presence of a 7SL sequence upstream has been shown to increase *Alu *transcription [27]. However, RepeatMasker [31] analysis indicates that the upstream flanking sequence of this *Alu *element is not a 7SL sequence but rather an ancient DNA transposon classified as a hAT-Charlie. Therefore, an alternative theory is that the 5'TATAAAAA3' sequence is not a functional TATA box but rather a simple variant of the classical TTTTAAAA or TTAAAA endonuclease cleavage site of L1 that is considered the preferred insertion site for *Alu *elements [32,33]. The potential role of this upstream sequence in the retrotransposition ability of this *Alu *element is not clear. However, the rhesus macaque genome (rheMac2) has a different sequence at this homologous position, 5'TATCAAAA3', and also does not have the *Alu *insertion.

Another factor determined to be critical for *Alu *replication is the structural integrity of the internal RNA Pol III promoter A and B boxes [4,7,34]. Two protein components of the signal recognition particle (SRP 9p and 14p) are believed to bind to specific *Alu *sequences during L1-mediated TPRT [35] and these SRP9/14 binding sites in the left monomer are required for *Alu *activity [4,7,34]. Bennett and colleagues demonstrated experimentally that mutating the SRP9/14 binding site in the left monomer reduced *Alu *mobilization efficiency to only 12% of normal, whereas a similar mutation in the right monomer, while also decreasing SRP9/14 binding, produced only a moderate decrease in retrotransposition efficiency, suggesting that an intact left monomer is more important for *Alu *mobilization [7]. The Chr7 *Alu *reported here has a completely conserved left monomer in orangutans, even though it is relatively old.

The degree of sequence variation between a candidate *Alu *'master' element and a known polymorphic subfamily has also been reported to impact mobilization efficiency [7]. The O:Chr7 progenitor *Alu *element in the orangutan appears to have only two random substitutions that do not appear evident in its proposed progeny, and both variants are located in the right monomer. The first single nucleotide substitution is a CpG mutation at position 154 that is present in the ponAbe2 genome assembly (Figure 2) but does not completely segregate in all the orangutans we sequenced at this locus. Bornean orangutan KB5405 exhibited the original cytosine nucleotide at this position in all the clones we sequenced (Additional file 1: Figure S1). It is known that about 30% of all CpG sites reside within *Alu *elements [36] and that CpG sites have six to ten times faster mutation rates than non-CpG sites [37-39], increasing the potential for independently occurring random mutation events. The second single nucleotide substitution in O:Chr7 is a relatively recent C to T transition at position 247 that also does not completely segregate in all the orangutans we tested. It is completely absent from the Bornean orangutans (they all have the ancestral cytosine nucleotide) and remains polymorphic with an allele frequency of 50% in the tested Sumatran orangutans (Additional file 1: Figure S1). The overall lack of sequence divergence (< 1%) between the ancestral O:Chr7 *Alu *element and the consensus sequence of the young polymorphic *Alu*Ye5b5_*Pongo *subfamily in orangutan strongly supports its candidacy as the founder element from which the young subfamily derived.

The human Chr7 *Alu *element appears to have three substitutions that are not present in the H:Chr3 *Alu *insertion, a CpG mutation at position 239 and two transversions: A to T at position 94 and T to G at position 173 (Figure 2). However, it is entirely possible, even probable, that all three substitutions occurred after the insertion in the H:Chr3 locus. The presence of a guanine residue at position 173 coincides with the consensus sequence of the human *Alu*Yf5 subfamily [31] and represents a single difference from the *Alu*Ye5 subfamily consensus sequence [22,23]. Although the *Alu*Yf5 subfamily was likely mobilizing in primate genomes around the same time, based on the sequence structure of the locus it is unlikely that the human Chr7 *Alu *insertion contributed to the proliferation of this subfamily.

Another factor influencing *Alu *activity is the distance of the Pol III TTTT termination signal from the 3' end of the element. Comeaux and colleagues used *Alu *A tail constructs to experimentally determine the effect of various 3' end lengths on *Alu *mobilization [4]. They reported a strong decrease in *Alu *retrotransposition ability even with little sequence between the end of the A tail and the Pol III terminator. The Chr7 *Alu *element reported here has the Pol III transcription terminator (TTTT) in the 3' TSD immediately following the A tail, a characteristic associated with mobilization ability.

The length of the poly(A) tail has also been reported to influence *Alu *retrotransposition activity, with longer A-tails free of nucleotide substitutions being more characteristic of young active source elements [8,40]. Mobilization ability in an *ex vivo *assay is reportedly very limited with a poly(A) tail less than 15 bp (base pairs) and increases thereafter to plateau at about 50 bp [40]. Under endogenous conditions, there appears to be only a modest benefit to *Alu *retrotransposition efficiency once the poly(A) tail exceeds about 20 bp [4]. The human Chr7 *Alu *element has a poly(A) tail length of 26 bp with two nucleotide substitutions and the O:Chr7 *Alu *element has a poly(A) tail length of 27 bp with three nucleotide substitutions (Additional file 1: Figure S1). These poly(A) tail lengths are consistent with possible activity. In addition, the youngest orangutan *Alu *progeny element in this study (O:Chr17:56932716) displays a perfect 30 bp poly(A) tail (Additional file 3), consistent with the literature. Because older *Alu *elements tend to have less pristine poly(A) tails compared to younger elements, Comeaux and colleagues used *Alu *A tail constructs to experimentally determine the impact of A tail disruptions on retrotransposition efficiency [4]. They demonstrated that nucleotide disruptions within the poly(A) tail are not created equal, in that adenine to thymine disruptions were relatively well tolerated with regard to maintaining the integrity of *Alu *mobilization, whereas nucleotide disruptions by cytosine or guanine resulted in greater impairment to retrotransposition efficiency [4]. The human Chr7 *Alu *element has a cytosine A tail disruption after 14 A-residues and a second one after 20 A-residues, perhaps impairing its current ability to propagate new copies. The O:Chr7 A tail has acquired a double cytosine (CC) mutation after only 10 A-residues and a third after only 16 A-residues (Additional file 1: Figure S1). These mutations may have rendered this ancestral *Alu *source element currently inactive.

With the exception of poly(A) tail disruptions, which may have occurred relatively recently, the ancestral Chr7 *Alu *insertion reported here possesses many of the classical hallmarks of being retrotranspositionally competent. *Alu *elements, like other retrotransposons, typically acquire nucleotide substitutions at a neutral rate after insertion [41]. Consequently, older elements tend to have a greater number of mutations (on average) than younger insertions. These acquired nucleotide substitutions often alter their ability to mobilize [4]. The Chr7 *Alu *reported here has remained highly conserved, especially in orangutans, even though it is approximately 16 million years old. This prompted us to speculate whether this element avoided the typical accumulation of random mutations because of its location in the *DGKB *gene or simply by chance.

According to the UCSC Genome Browser [18,19] Gene Sorter function, the human *DGKB *gene is about 693 kb long (693,643 bp), of which only 2,415 bp is coding sequence (< 0.35%), comprising 804 amino acids distributed among 25 exons. Twenty-four introns make up the vast majority of the gene sequence. Zhang and colleagues [42] recently reported that, although *Alu *density is quite low in exons of genes (selected against), the *Alu *density in introns of genes is similar to the *Alu *density in intergenic regions of the genome, suggesting a similar selective pressure (essentially neutral). The *DGKB *gene has no *Alu *element insertions within its exons or promoter regions, but has 113 *Alu *element insertions within introns as identified by the TranspoGene database [43,44]. Of the 113 *Alu *elements located within the gene, 19 were identified as *Alu*Y or younger, including the *Alu *element from this study, which is located in intron 20 of 24. We screened the other 18 *Alu*Y elements to find those with the same species distribution to the *Alu *element in this study and therefore expected to be of similar age. We selected seven full-length (> 275 bp) *Alu*Y insertions from the *DGKB *gene that are shared in human, chimpanzee and orangutan, while absent from the rhesus macaque genome. We constructed a sequence alignment of these *Alu *elements from hg18 and ponAbe2 to compare their percent divergence from the consensus sequence compared to our element. The percent divergence of the human and orangutan *Alu*Y insertions was 6.1 ± 1.4 and 8.6 ± 1.4, respectively, compared to 3.6 and 3.3 respectively for the *Alu *element in this study (Additional file 4). Although this does not conclusively prove that the location of the *Alu *element in the *DGKB *gene has no effect, it does suggest that merely being present within a gene as opposed to within an intergenic sequence does not necessarily offer an *Alu *element protection against age-associated degradation. It also confirms that the *Alu *element in this study is unusually pristine for its age, a characteristic associated with mobilization ability. The reason for this, if not simply by chance alone, is not clear. The structure of this *Alu *element and its sequence evolution in multiple species is not consistent with a gene conversion event, nor is there any evidence of differential selection. It is possible that the *Alu *element is located in a more protected hypomethylated environment that is not similar to the other *Alu *element insertions we examined in the same gene, one of which was in the same intron. But to determine this would require a more comprehensive study of the *DGKB *gene and its evolution,

We have estimated the Chr7 *Alu *insertion in this study to be about 16 million years old and concluded that this insertion was most likely a member of the *Alu*Ye lineage upon its insertion. In order to determine if this subfamily was actively mobilizing during the estimated time period, we examined data from a previous analysis of the *Alu*Ye lineage in which Salem and colleagues used PCR to determine the species distribution of 118 *Alu*Ye5 subfamily members [45]. Of these, about 32% (38 of 118) exhibited the same species distribution to the *Alu *element in this study while another 21% of the subfamily members (25 of 118) represented even older insertions that were also shared with siamang (present in human, chimpanzee, gorilla, orangutan and siamang). The remaining *Alu*Ye5 elements represented younger insertions, present in human, chimpanzee and gorilla but absent from orangutan (33%), present in human and chimpanzee only (7%) or were human-specific insertions (7%). The findings of this previous study demonstrate that the *Alu *subfamily from which the Chr7 *Alu *insertion in this study is derived was actively propagating during the estimated time of its insertion. Moreover, in the orangutan lineage, the Chr7 ancestral *Alu *element underwent a hierarchical accumulation of multiple post-insertion diagnostic substitutions in the right arm, while also failing to accumulate the more likely random variants over the same evolutionary time period. It is inconceivable that by chance alone these post-insertion diagnostic substitutions just happen to match the young polymorphic *Alu*Ye5b5_*Pongo *elements in the orangutan, and that it is the only element identified in the orangutan genome to do so.

Our findings are consistent with a modified 'master gene' model of *Alu *amplification, or 'stealth model' for the expansion of lineage-specific *Alu *subfamilies [46]. It has been well established that *Alu *subfamilies > 20 million years old still have active members in primate genomes [45,47]. Studies of human *Alu *subfamilies have demonstrated that about 15% of subfamily members are active as secondary source elements [48], leading to a complex bush-like expansion of lineage-specific *Alu *subfamilies [48,49]. Under the stealth-driver model, an *Alu *lineage can remain quiescent for millions of years while maintaining low levels of retrotransposition activity to allow the lineage to persist over time [46,50]. In the case of the orangutan genome, the relative quiescence of *Alu *retrotransposition in the last several million years may have resulted from a population bottleneck or other demographic factors impacting their genomic architecture, and effectively disrupting the primary master-driver elements [51]. In this scenario, the survival of an *Alu *lineage would require the persistence of a few very old active copies that fortuitously avoided mutational decay, slowly giving rise to more recent active daughter elements. The recent expansion of the *Alu*Ye5b5_*Pongo *subfamily in orangutan is consistent with the existence of such a backseat driver. Conversely, in the human genome, the expansion of *Alu *from the Chr7 locus has remained quite limited, possibly due to the overall abundance of more robust *Alu *systems over the same evolutionary time period.

## Conclusions

The objective of this study was to search for *Alu *source elements in the orangutan genome, against a background of relatively sparse young insertions. Using the youngest insertions as templates, we identified a much older, but nearly identical *Alu *insertion on Chr7. We demonstrated that this insertion is shared among great apes and possesses classical hallmarks of mobilization ability. We provided evidence for the concurrent evolution of lineage-specific insertion events from this source element in the orangutan and human genomes. Accumulated mutations within the poly(A) tails of these stealth drivers may have forced their eventual retirement. However, the sequential propagation of this *Alu *lineage in the orangutan genome, ongoing at a very low rate over millions of years of evolution and then recently sprouting young offspring, supports our finding of an ancient backseat driver of *Alu *element expansion.

## Methods

### Orangutan-specific *Alu *subfamilies

Of the orangutan-specific *Alu *subfamilies, three were determined to be the youngest based on their divergence from their respective consensus sequences. Using the standardized nomenclature for *Alu *repeats as a guideline [16], these three subfamilies have been termed *Alu*Yc1a5_*Pongo, Alu*Ye5a2_*Pongo *and *Alu*Ye5b5_*Pongo*. The consensus sequences are available as Additional file 5. The naming convention is as follows: The *Alu*Yc1a5_*Pongo *subfamily shares the same 12 bp deletion as the human *Alu*Yc and Rhesus *Alu*YRb lineages and likely derived from a common ancestor. In addition, it has six other mutations, only one of which is shared in the human and rhesus lineages, while the other five mutations seem unique to the orangutan lineage. The *Alu*Ye5a2_*Pongo *subfamily shares all the same insertions and deletions (indels) and substitutions of the human *Alu*Ye5 subfamily [22,23], and has two additional transition mutations, hence 'a2'. Following this same naming convention, the *Alu*Ye5b5_*Pongo *subfamily also shares all the indels and substitutions of the human *Alu*Ye5 subfamily, but has five additional mutations, none of which are the same as the two characteristic of *Alu*Ye5a2_*Pongo *described above, hence 'b5'.

### Computational data collection

We computationally searched the ponAbe2 (July 2007) orangutan genome assembly on the UCSC Genome Browser [18,19] using BLAT [17] for the closest matches to each of the three polymorphic *Alu *subfamily consensus sequences. Each locus was evaluated for species specificity using the UCSC Genome Browser and sequences were retrieved and aligned using BioEdit [52] or MegAlign with the ClustalW algorithm (DNASTAR, Inc. Version 5.0 for Windows).

### DNA

The DNA panel used for PCR analysis of candidate *Alu *loci included human (*Homo sapiens)*, bonobo chimpanzee *(Pan paniscus)*, common chimpanzee *(Pan troglodytes)*, lowland gorilla *(Gorilla gorilla)*, Bornean orangutan *(Pongo pygmaeus)*, Sumatran orangutan *(Pongo abelii)*, siamang *(Hylobates syndactylus)*, white-handed gibbon *(Hylobates lar)*, red-cheeked gibbon *(Hylobates gabriellai)*, African green monkey *(Chlorocebus aethiops) *and rhesus macaque *(Macaca mulatta)*. Orangutan DNA samples (n = 37) were obtained from the Coriell Institute for Medical Research, Camden, NJ (n = 6), the San Diego Frozen Zoo (n = 19) and the National Institutes of Health National Cancer Institute (n = 12). A list of these DNA samples is available in Additional file 2; DNA samples. The human-specific locus on chromosome 3 was analyzed in order to determine if it was polymorphic within a DNA panel of 80 individuals (20 African Americans, 20 Asians, 20 Europeans and 20 South Americans) obtained from the Coriell Institute for Medical Research, Camden, NJ (Additional file 2; DNA samples).

### PCR primer design

Primers were manually designed in conserved regions of the orangutan, human, chimpanzee and rhesus macaque flanking sequences in order to increase the likelihood of successful amplification across various species under consideration of RepeatMasker output files [31]. For each locus, sequences were retrieved from UCSC [18,19] and aligned using BioEdit [52] or MegAlign with the ClustalW algorithm. Each primer was checked with BLAT [17] to insure amplification of a single locus. In addition, a virtual PCR was performed for each locus using the *in silico *function of BLAT [17] in order to receive an estimated PCR product size for the empty (no insertion) and the filled size (insertion present). Primers were obtained from Sigma Aldrich (Woodlands, TX, USA).

### PCR analysis

PCR amplifications were performed in 25 μL reactions containing 15 to 50 ng of template DNA, 200 nM of each oligonucleotide primer, 1.5 to 3.0 mM MgCl_2_, 10× PCR buffer (50 mM KCl, 10 mM TrisHCl; pH 8.4), 0.2 mM deoxyribonucleotide triphosphates and 1 to 2 U *Taq *DNA polymerase. PCR reactions were performed under the following conditions: initial denaturation at 94°C for 60 seconds, followed by 32 cycles of denaturation at 94°C for 30 seconds, 30 seconds at primer annealing temperature (Additional file 2; PCR primers and conditions), and extension at 72°C for 30 seconds. PCRs were terminated with a final extension at 72°C for 2 minutes. Fractionation of 20 μL of each PCR product was performed in a horizontal gel chamber on a 2% agarose gel containing 0.2 μg/mL ethidium bromide for 60 to 70 minutes at 175 V. UV-fluorescence was used to visualize the DNA fragments.

### Cloning and sequencing

The original PCR primer pair used to screen the Chr7 Alu insertion resulted in a filled size of about 707 bp (shown in Figure 1). In an effort to improve the efficiency of cloning and sequencing, a second set of PCR primers was designed to produce a smaller amplicon of about 475 bp (Additional file 2; PCR Primers). PCR fragments were gel purified using Wizard SV gel purification (Promega Corporation, Madison, WI, USA, catalog A9282) and cloned using TOPO TA cloning kits for sequencing (Invitrogen Corporation, Carlsbad, CA, USA, catalog K4575-40). A total of four to eight clones from each sample were sequenced using Sanger sequencing on an ABI 3130xl genome analyzer (Applied Biosystems, Inc., Foster City, CA, USA). Sequence quality was evaluated using ABI software Sequence Scanner v1.0. Sequences were analyzed using DNASTAR version 5.0 for windows and aligned using MegAlign. Sequence alignment figures were constructed by selecting the 'view alignment report' option in MegAlign, following manual formatting of 'alignment report contents' under Options. The output was saved as a text file, followed by manual refinement and labeling in Microsoft Word for windows.

## Abbreviations

bp: base pair; *DGKB*: diacylglycerol kinase beta enzyme gene; Chr7: chromosome 7; kb: kilobase; LINE: long interspersed element; O:Chr: orangutan chromosome; PCR: polymerase chain reaction; Pol III: RNA polymerase III; SRP: signal recognition particle; TPRT: target-primed reverse transcription; TSD: target site duplication; UCSC: University of California Santa Cruz.

## Competing interests

The authors declare that they have no competing interests.

## Authors' contributions

MKK, JAW and MAB designed the research and wrote the paper; MKK, JAW and CPM conducted the experiments; AFAS, RH, BU and MKK performed the repeat analysis of the ponAbe2 genome assembly; OAR provided DNA samples and edited the manuscript. All authors read and approved the final manuscript.

## Description of additional data files

The following additional data files are available with the online version of this paper. Additional file 1 is a sequence alignment report of the chromosome 7 *Alu *insertion with the flanking sequence for multiple orangutan individuals and other primates obtained by Sanger sequencing of PCR amplicons. Additional file 2 is a series of tables listing the PCR primers and conditions, genotypes and allele frequencies, and DNA samples. Additional file 3 provides FASTA output for potential secondary source elements in orangutan. Additional file 4 is the TranspoGene database [44] output for the DGBK gene. Additional file 5 provides the *Alu *subfamily consensus sequences for the three youngest orangutan-specific subfamilies.

## Authors' information

CPM conducted experiments for this project in the Department of Biological Sciences, LSU-Baton Rouge as a participant in the Louisiana Biomedical Research Network (LBRN) while completing a degree in the School of Biological Sciences, Louisiana Tech University-Ruston, LA. CPM is currently a graduate student in the Department of Molecular and Cellular Physiology at LSUHSC-Shreveport.

## Supplementary Material

Additional file 1 **Figure S1 and Table S1**. This file contains a sequence alignment report of the Chr7 *Alu *insertion with flanking sequence for multiple orangutan individuals and other primates obtained by Sanger sequencing of PCR amplicons and a list the DNA samples used to construct the alignment. Click here for file

Additional file 2 **Table S2**. This file contains a series of tables listing the PCR primers and conditions, genotypes and allele frequencies, and DNA samples used in the study. Click here for file

Additional file 3 **Table S2**. This file provides FASTA output for three of the potential secondary source elements in the orangutan from the [ponAbe2] genome assembly and Additional file 2: Table S2. Click here for file

Additional file 4 **This file contains the TranspoGene database **[42]** output for the DGBK gene**. Click here for file

Additional file 5 **This file provides the *Alu *subfamily consensus sequences for the three youngest orangutan-specific subfamilies**. Click here for file
