# Supplementary material for: Orangutan Alu quiescence reveals possible source element: support for ancient backseat drivers
Source: Mob DNA. 2012 Apr 30;3:8. doi: 10.1186/1759-8753-3-8 (PMC3357318; doi:10.1186/1759-8753-3-8)
Supplement: Additional file 1 — Figure S1 and Table S1. This file contains a sequence alignment report of the Chr7 Alu insertion with flanking sequence for multiple orangutan individuals and other primates obtained by Sanger sequencing of PCR amplicons and a list the DNA samples used to construct the alignment. [file 1759-8753-3-8-S1.PDF]

Figure S1

```
AluYconsensus      - - - - - - - - - - - - - - - - - - - - - - - - - - - - - - - - - - - - - - - - - - - - - - - - - -  0
Human-BLAT         T G G T T A T T T G T T T T G G G G A A G T A A C A T T T T G C T T A A T A G T G G T T C A A A A T  50
Chimp-BLAT         T G G T T A T T T G T T C T G G G G A A G T A A C A T T T T G C T T A A T A G T G G C T C A A A A T  50
Pan paniscus(I)    T G G T T A T T T G T T C T G G G G A A G T A A C A T T T T G C T T A A T A G T G G T T C A A A A T  50
Pan paniscus(C)    T G G T T A T T T G T T C T G G G G A A G T A A C A T T T T G C T T A A T A G T G G T T C A A A A T  50
Gorilla gorilla    T G G T T A T T T G T T C T G G G G A A G T A A C A T T T T G C T C A A T A G T G G T T C A A A A T  50
Orangutan-BLAT     T G G T T A T T T G T T T T G G G G A A G T A A C A T T T T G C T T A A T A G T G G T T C A A A A T  50
Susie Ref.         T G G T T A T T T G T T T T G G G G A A G T A A C A T T T T G C T T A A T A G T G G T T C A A A A T  50
GM06213A           T G G T T A T T T G T T T T G G G G A A G T A A C A T T T T G C T T A A T A G T G G T T C A A A A T  50
GM04272A           T G G T T A T T T G T T C T G G G G A A G T A A C A T T T T G C T T A A T A G T G G T T C A A A A T  50
NG12256            T G G T T A T T T G T T T T G G G G A A G T A A C A T T T T G C T T A A T A G T G G T T C A A A A T  50
NG06209            T G G T T A T T T G T T T T G G G G A A G T A A C A T T T T G C T T A A T A G T G G T T C A A A A T  50
AG05252A           T G G T T A T T T G T T T T G G G G A A G T A A C A T T T T G C T T A A T A G T G G T T C A A A A T  50
KB4204             T G G T T A T T T G T T T T G G G G A A G T A A C A T T T T G C T T A A T A G T G G T T C A A A A T  50
KB5404             T G G T T A T T T G T T T T G G G G A A G T A A C A T T T T G C T T A A T A G T G G T T C A A A A T  50
KB5405             T G G T T A T T T G T T T T G G G G A A G T A A C A T T T T G C T T A A T A G T G G T T C A A A A T  50

AluYconsensus      - - - - - - - - - - - - - - - - - - - - - - - - - - - - - - - - - - - - - - - - - - - - - - - - -  0
Human-BLAT         T G G T G T T C G T T G T C A T C A A A T C A G T T A C A C A T A C A C A A G C T G T A T A A A A A 100
Chimp-BLAT         T G G T G T T C G T T G T C A T C A A A T C A G T T A C A C A T A C A C A A G C T G T A T A A A A A 100
Pan paniscus(I)    T G G T G T T C G T T G T C A T C A A A T C A G T T A C A C A T A C A C A A G C T G T A T A A A A A 100
Pan paniscus(C)    T G G T G T T C G T T G T C A T C A A A T C A G T T A C A C A T A C A C A A G C T G T A T A A A A A 100
Gorilla gorilla    T G G T G T T C G T T G T C A T C A A A T C A G T T A C A C A T A C A C A A G C T G T A T A A A A A 100
Orangutan-BLAT     T G G T G T T C C T T G T C A T C A A A T C A G T T A C A T A T A C A C A A G C T G T A T A A A A A 100
Susie Ref.         T G G T G T T C C T T G T C A T C A A A T C A G T T A C A T A T A C A C A A G C T G T A T A A A A A 100
GM06213A           T G G T G T T C C T T G T C A T C A A A T C A G T T A C A T A T A C A C A A G C T G T A T A A A A A 100
GM04272A           T G G T G T T C C T T G T C A T C A A A T C A G T T A C A T A T A C A C A A G C T G T A T A A A A A 100
NG12256            T G G T G T T C C T T G T C A T C A A A T C A G T T A C A T A T A C A C A A G C T G T A T A A A A A 100
NG06209            T G G T G T T C C T T G T C A T C A A A T C A G T T A C A T A T A C A C A A G C T G T A T A A A A A 100
AG05252A           T G G T G T T C C T T G T C A T C A A A T C A G T T A C A T A T A C A C A A G C T G T A T A A A A A 100
KB4204             T G G T G T T C C T T G T C A T C A A A T C A G T T A C A T A T A C A C A A G C T G T A T A A A A A 100
KB5404             T G G T G T T C C T T G T C A T C A A A T C A G T T A C A T A T A C A C A A G C T G T A T A A A A A 100
KB5405             T G G T G T T C C T T G T C A T C A A A T C A G T T A C A T A T A C A C A A G C T G T A T A A A A A 100
                                       -----------------+-------------------+-------------------+-------------------+-
                                                        10                  20                  30                  40
                                       -----------------+-------------------+-------------------+-------------------+-
AluYconsensus      - - - - - - - - - - G G C C G G G C G C G G T G G C T C A C G C C T G T A A T C C C A G C A C T T T  40
Human-BLAT         C A G G T C T G T T G A C C G G G C G C G G T G G C T C A C G C C T G T A A T C C C A G C A C T T T 150
Chimp-BLAT         C A G G T C T G T T G A C C G G G T G C G G T G G C T C A C G C G T G T A A T C C C A G C A C T T T 150
Pan paniscus(I)    C A G G T C T G T T G A C C G G G T G C G G T G G C T C A C G C G T G T A A T C C C A G C A C T T T 150
Pan paniscus(C)    C A G G T C T G T T G A C C G G G T G C G G T G G C T C A C G C G T G T A A T C C C A G C A C T T T 150
Gorilla gorilla    C A G G T C T G T T G A C C G G G C G C G G T G G C T C A C G C C T G T A A T C C C A G C A C T T C 150
Orangutan-BLAT     T A G G T C T G T T G G C C G G G C G C G G T G G C T C A C G C C T G T A A T C C C A G C A C T T T 150
Susie Ref.         T A G G T C T G T T G G C C G G G C G C G G T G G C T C A C G C C T G T A A T C C C A G C A C T T T 150
GM06213A           T A G G T C T G T T G G C C G G G C G C G G T G G C T C A C G C C T G T A A T C C C A G C A C T T T 150
GM04272A           T A G G T C T G T T G G C C G G G C G C G G T G G C T C A C G C C T G T A A T C C C A G C A C T T T 150
NG12256            T A G G T C T G T T G G C C G G G C G C G G T G G C T C A C G C C T G T A A T C C C A G C A C T T T 150
NG06209            T A G G T C T G T T G G C C G G G C G C G G T G G C T C A C G C C T G T A A T C C C A G C A C T T T 150
AG05252A           T A G G T C T G T T G G C C G G G C G C G G T G G C T C A C G C C T G T A A T C C C A G C A C T T T 150
KB4204             T A G G T C T G T T G G C C G G G C G C G G T G G C T C A C G C C T G T A A T C C C A G C A C T T T 150
KB5404             T A G G T C T G T T G G C C G G G C G C G G T G G C T C A C G C C T G T A A T C C C A G C A C T T T 150
KB5405             C A G G T C T G T T G G C C G G G C G C G G T G G C T C A C G C C T G T A A T C C C A G C A C T T T 150
```

```
                                      50                  60                  70                  80                  90
                        -----------------+-------------------+-------------------+-------------------+-------------------+-
AluYconsensus     G G G A G G C C G A G G C G G G C G G A T C A C G A G G T C A G G A G A T C G A G A C C A T C C T G  90
Human-BLAT        G G G A G G C C G A G G C G G G C G G A T C A C G A G G T C A G G A G A T C G A G A C C A T C C T G 200
Chimp-BLAT        G G G C G G C C G A G G C G G G C G G A T C A C G A G G T C A G G A G A T C G A G A C C A T C C T G 200
Pan paniscus(I)   G G G C G G C C G A G G C G G G C G G A T C A C G A G G T C A G G A G A T C G A G A C C A T C C T G 200
Pan paniscus(C)   G G G C G G C C G A G G C G G G C G G A T C A C G A G G T C A G G A G A T C G A G A C C A T C C T G 200
Gorilla gorilla   C G G A G G C C G A G G C G G G C G G A T C A C G A G G T C A G G A G A T C G A G A C C A T C C T G 200
Orangutan-BLAT    G G G A G G C C G A G G C G G G C G G A T C A C G A G G T C A G G A G A T C G A G A C C A T C C T G 200
Susie Ref.        G G G A G G C C G A G G C G G G C G G A T C A C G A G G T C A G G A G A T C G A G A C C A T C C T G 200
GM06213A          G G G A G G C C G A G G C G G G C G G A T C A C G A G G T C A G G A G A T C G A G A C C A T C C T G 200
GM04272A          G G G A G G C C G A G G C G G G C A G A T C A C G A G G T C A G G A G A T C G A G A C C A T C C T G 200
NG12256           G G G A G G C C G A G G C G G G C G G A T C A C G A G G T C A G G A G A T C G A G A C C A T C C T G 200
NG06209           G G G A G G C C G A G G C G G G C G G A T C A C G A G G T C A G G A G A T C G A G A C C A T C C T G 200
AG05252A          G G G A G G C C G A G G C G G G C G G A T C A C G A G G T C A G G A G A T C G A G A C C A T C C T G 200
KB4204            G G G A G G C C G A G G C G G G C G G A T C A C G A G G T C A G G A G A T C G A G A C C A T C C T G 200
KB5404            G G G A G G C C G A G G C G G G C G G A T C A C G A G G T C A G G A G A T C G A G A C C A T C C T G 200
KB5405            G G G A G G C C G A G G C G G G C G G A T C A C G A G G T C A G G A G A T C G A G A C C A T C C T G 200
                                      100                 110                 120                 130                 140
                        -----------------+-------------------+-------------------+-------------------+-------------------+-
AluYconsensus     G C T A A C A C G G T G A A A C C C C G T C T C T A C T A A A A A T A C A A A A A A T T A G C C G G 140
Human-BLAT        G C T T A C A C G G T G A A A C C C C G T C T C T A C T A A A A A T A C A A A A A A T T A G C C T G 250
Chimp-BLAT        G C T A A C A C G G T G A A A C C C C G T C T C T A C T A A A A A T A C A A A A A A T T A G C C T G 250
Pan paniscus(I)   G C T A A C A C G G T G A A A C C C C G T C T C T A C T A A A A A T A C A A A A A A T T A G C C T G 250
Pan paniscus(C)   G C T A A C A C G G T G A A A C C C C G T C T C T A C T A A A A A T A C A A A A A A T T A G C C T G 250
Gorilla gorilla   G C T A A C A C G G T G A A A C C C C G T C T C T A C T A A A A A T A C A A A A A A T T A G C C T G 250
Orangutan-BLAT    G C T A A C A C G G T G A A A C C C C G T C T C T A C T A A A A A T A C A A A A A A T T A G C C T G 250
Suzie Ref.        G C T A A C A C G G C G A A A C C C C G T C T C T A C T A A A A A T A C A A A A A A T T A G C C T G 250
GM06213A          G C T A A C A C G G T G A A A C C C C G T C T C T A C T A A A A A T A C A A A A A A T T A G C C T G 250
GM04272A          G C T A A C A C G G T G A A A C C C C G T C T C T A C T A A A A A T A C A A A A A A T T A G C C T G 250
NG12256           G C T A A C A C G G T G A A A C C C C G T C T C T A C T A A A A A T A C A A A A A A T T A G C C T G 250
NG06209           G C T A A C A C G G T G A A A C C C C G T C T C T A C T A A A A A T A C A A A A A A T T A G C C T G 250
AG05252A          G C T A A C A C G G T G A A A C C C C G T C T C T A C T A A A A A T A C A A A A A A T T A G C C T G 250
KB4204            G C T A A C A C G G T G A A A C C C C G T C T C T A C T A A A A A T A C A A A A A A T T A G C C T G 250
KB5404            G C T A A C A C G G T G A A A C C C C G T C T C T A C T A A A A A T A C A A A A A A T T A G C C T G 250
KB5405            G C T A A C A C G G T G A A A C C C C G T C T C T A C T A A A A A T A C A A A A A A T T A G C C T G 250
                                      150                 160                 170                 180                 190
                        -----------------+-------------------+-------------------+-------------------+-------------------+-
AluYconsensus     G C G T G G T G G C G G G C G C C T G T A G T C C C A G C T A C T C G G G A G G C T G A G G C A G G 190
Human-BLAT        G C A A G G T G G C G G G C G C C T G T A G T C C C A G C T A C G C G G G A G G C T G A G G C A G G 300
Chimp-BLAT        G C A A G G T G G C G G G C G C C T G T A G T C C C A G C T C C T C G G G A G G C T G A G G C A G G 300
Pan paniscus(I)   G C A A G G T G G C G G G C G C C T G T A G T C C C A G C T C C T C G G G A G G C T G A G G C A G A 300
Pan paniscus(C)   G C A A G G T G G C G G G C G C C T G T A G T C C C A G C T C C T C G G G A G G C T G A G G C A G A 300
Gorilla gorilla   G C A A G G T G G C G G G C G C C T G T A G T C C C A G C T C C T C G G G A G G C T G A G G C A G G 300
Orangutan-BLAT    G C G A G G T G G C G G G T G C C T G T A G T C C C A G C T A C T C G G G A G G C T G A G G C A G G 300
Susie Ref.        G C G A G G T G G C G G G T G C C T G T A G T C C C A G C T A C T C G G G A G G C T G A G G C A G G 300
GM06213A          G C G A G G T G G C G G G T G C C T G T A G T C C C A G C T A C T C G G G A G G C T G A G G C A G G 300
GM04272A          G C G A G G T G G C G G G T G C C T G T A G T C C C A G C T A C T C G G G A G G C T G A G G C A G G 300
NG12256           G C G A G G T G G C G G G T G C C T G T A G T C C C A G C T A C T C G G G A G G C T G A G G C A G G 300
NG06209           G C G A G G T G G C G G G T G C C T G T A G T C C C A G C T A C T C G G G A G G C T G A G G C A G G 300
AG05252A          G C G A G G T G G C G G G T G C C T G T A G T C C C A G C T A C T C G G G A G G C T G A G G C A G G 300
KB4204            G C G A G G T G G C G G G T G C C T G T A G T C C C A G C T A C T C G G G A G G C T G A G G C A G G 300
KB5404            G C G A G G T G G C G G G T G C C T G T A G T C C C A G C T A C T C G G G A G G C T G A G G C A G G 300
KB5405            G C G A G G T G G C G G G C G C C T G T A G T C C C A G C T A C T C G G G A G G C T G A G G C A G G 300
```

```
                              200                 210                 220                 230                 240
                 -----------------+-----------------+-----------------+-----------------+-----------------+
AluYconsensus    A G A A T G G C G T G A A C C C - G G G A G G C G G A G C T T G C A G T G A G C C G A G A T C G C G 239
Human-BLAT       A G A A T G G C G T G A A C C C C A G G G G G C A G A G C T T G C A G T G A G C C G A G A T C G T G 350
Chimp-BLAT       A G A A T G G C G T G A A C C C C A G G G G G C A G A G C T T G C A G T G A G C C G A G A T C G C G 350
Pan paniscus(I)  A G A A T G G C G T G A A C C C C A G G G G G C A G A G C T T G C A G T G A G C C G A G A T C G C G 350
Pan paniscus(C)  A G A A T G G C G T G A A C C C C A G G G G G C A G A G C T T G C A G T G A G C C G A G A T C G C G 350
Gorilla gorilla  A G A A T G G C G T G A A C C C C A G G G G G C A G A G C T T G C A G T G A G C C G A G A T C G C G 350
Orangutan-BLAT   A G A A T G G C G T G A A C C C C A G G G G G C G G A G C C T G C A G T G A G C T G A G A T C G C G 350
Suzie Ref.       A G A A T G G C G T G A A C C C C A G G G G G C G G A G C C T G C A G T G A G C T G A G A T C G C G 350
GM06213A         A G A A T G G C G T G A A C C C C A G G G G G C G G A G C C T G C A G T G A G C T G A G A T C G C G 350
GM04272A         A G A A T G G C G T G A A C C C C A G G G G G C G G A G C T T G C A G T G A G C T G A G A T C G C G 350
NG12256          A G A A T G G C G T G A A C C C C A G G G G G C G G A G C C T G C A G T G A G C T G A G A T C G C G 350
NG06209          A G A A T G G C G T G A A C C C C A G G G G G C G G A G C C T G C A G T G A G C T G A G A T C G C G 350
AG05252A         A G A A T G G C G T G A A C C C C A G G G G G C G G A G C C T G C A G T G A G C T G A G A T C G C G 350
KB4204           A G A A T G G C G T G A A C C C C A G G G G G C G G A G C C T G C A G T G A G C T G A G A T C G C G 350
KB5404           A G A A T G G C G T G A A C C C C A G G G G G C G G A G C C T G C A G T G A G C T G A G A T C G C G 350
KB5405           A G A A T G G C G T G A A C C C C A G G G G G C G G A G C C T G C A G T G A G C C G A G A T C G T G 350

                              250                 260                 270                 280                 290
                 -----------------+-----------------+-----------------+-----------------+-----------------+
AluYconsensus    C C A C T G C A C T C C A G C C T G G G C G A C A G A G C G A G A C T C C G T C T C A A A A A A     287
Human-BLAT       C C A C T G C A C T C C A G C C T G G G C G A T A G - - C G A G A C T C C G T C T C A A A A A A A A A 398
Chimp-BLAT       C C A C T G C A C T C T A G C C T G G G T G A T A G - - C G A G A C T C C G T C T C A A A A A A A A A 398
Pan paniscus(I)  C C A C T G C A C T C C A G C C T G G G T G A T A G - - C G A G A C T C C G T C T C A A A A A A A A A 398
Pan paniscus(C)  C C A C T G C A C T C T A G C C T G G G T G A T A G - - C G A G A C T C C G T C T C A A A A A A A A A 398
Gorilla gorilla  C C A C T G C A C T C T A G C C T G G G T G A T A G - - C G A G A C T C C G T C T C A A A A A A A A A 398
Orangutan-BLAT   C C A C T G T A C T C C A G C C T G G G C G A T A G - - C G A G C C T C C G T C T C A A A A A A A A A 398
Suzie Ref.       C C A C T G T A C T C C A G C C T G G G C G A T A G - - C G A G C C T C C G T C T C A A A A A A A A A 398
GM06213A         C C A C T G T A C T C C A G C C T G G G C G A T A G - - C G A G C C T C C G T C T C A A A A A A A A A 398
GM04272A         C C A C T G C A C T C C A G C C T G G G C G A T A G - - C G A G C C T C C G T C T C A A A A A A A A A 398
NG12256          C C A C T G C A C T C C A G C C T G G G C G A T A G - - C G A G C C T C C G T C T C A A A A A A A A A 398
NG06209          C C A C T G T A C T C C A G C C T G G G C G A T A G - - C G A G C C T C C G T C T C A A A A A A A A A 398
AG05252A         C C A C T G C A C T C C A G C C T G G G C G A T A G - - C G A G C C T C C G T C T C A A A A A A A A A 398
KB4204           C C A C T G C A C T C C A G C C T G G G C G A T A G - - C G A G C C T C C G T C T C A A A A A A A A A 398
KB5404           C C A C T G C A C T C C A G C C T G G G C G A T A G - - C G A G C C T C C G T C T C A A A A A A A A A 398
KB5405           C C A C T G C A C T C C A G C C T G G G C G A T A G - - C G A G A C T C C G T C T C A A A A A A A A A 398

AluYconsensus                                                                                                        287
Human-BLAT       A A A A A C A A A A A C A A A A A A - - - - C A G G T C T G T T T T G G C C C T C A G G T T A T A A 444
Chimp-BLAT       A A A A A A A A A A A A A A A A A A A A A A C A G G T C T G T T T T G G C C C T C A G G T T A T A A 448
Pan paniscus(I)  A A A A A A A A A A A A A A A A - - - - - - C A G G T C T G T T T T G G C C C T C A G G T T A T A A 442
Pan paniscus(C)  A A A A A A A A A A A A A A A A A A A - - A C A G G T C T G T T T T G G C C C T C A G G T T A T A A 446
Gorilla gorilla  A C A A A - A A A A A A A A A A - - - - - - C A G G T C T G T T T T G G C C C T C A G G T T A T A A 441
Orangutan-BLAT   A A C C A A A A A A C A A A A A A A A - - - C A G G T C T G T T T T G G C C C T C A G G T T A T A A 445
Suzie Ref.       A A C C A A A A A A C A A A A A A A A - - - C A G G T C T G T T T T G G C C C T C A G G T T A T A A 445
GM06213A         A A C C A A A A A A C A A A A A A A A - - - C A G G T C T G T T T T G G C C C T C A G G T T A T A A 445
GM04272A         A A C C A A A A A A C A A A A A A A A - - - C A G G T C T G T T T T G G C C C T C A G G T T A T A A 445
NG12256          A A C C A A A A A A C A A A A A A A A - - - C A G G T C T G T T T T G G C C C T C A G G T T A T A A 445
NG06209          A A C C A A A A A A C A A A A A A A A - - - C A G G T C T G T T T T G G C C C T C A G G T T A T A A 445
AG05252A         A - C C A A A A A A C A A A A A A A A - - - C A G G T C T G T T T T G G C C C T C A G G T T A T A A 444
KB4204           A A C C A A A A A A C A A A A A A A A - - - C A G G T C T G T T T T G G C C C T C A G G T T A T A A 445
KB5404           A A C C A A A A A A C A A A A A A A A - - - C A G G T C T G T T T T G G C C C T C A G G T T A T A A 445
KB5405           A - C C A A A A A A C A A A A A A A A - - - C A G G T C T G T T T T G G C C C T C A G G T T A T A A 444
```

Figure S1. Sequence Alignment Report of Chr7 shared *Alu* insertion and flanking sequence. The TATA box-like sequence is conserved in all the species sequenced at this locus and is proximal to the 5' end of the *Alu* insertion (highlighted in blue). The Pol III transcription termination signal (TTTT) is also conserved in all species (highlighted in red) immediately after the 3' target site duplication (TSD). TSDs are highlighted in teal. Highlighted in pink are mutations different from the *Alu*Y subfamily consensus sequence that are shared by all investigated species and are all located in the right monomer of the element following the middle A-rich region. These coincide with diagnostic nucleotides of the *Alu*Ye5 subfamily. Orangutan-specific substitutions are highlighted in yellow. The *Homininae*-specific substitutions are highlighted in green. Chimpanzee / gorilla mutations are highlighted in brown and random mutations are shown in gray.

Table S1

DNA sequences in Alignment Report							
	**Species Names**	**Common Names**	**Origin**	**ID number**	**5' TATA box**	**5' TSD**	**3' TSD & termination signal**
1	*Homo sapiens*	Human	hg18	N/A	TATAAAAA	CAGGTCTGTT	CAGGTCTGTTTT
2	*Pan troglodytes*	Chimpanzee	panTro2	NS06006	TATAAAAA	CAGGTCTGTT	CAGGTCTGTTTT
3	*Pan paniscus*	Bonobo	IPBIR	PR00661	TATAAAAA	CAGGTCTGTT	CAGGTCTGTTTT
4	*Pan paniscus*	Bonobo	Coriell	AG05253	TATAAAAA	CAGGTCTGTT	CAGGTCTGTTTT
5	*Gorilla gorilla*	Lowland gorilla	Coriell	AG05251	TATAAAAA	CAGGTCTGTT	CAGGTCTGTTTT
6	*Pongo pygmaeus abelii*	Sumatran Orangutan	ponAbe2	PR01109 ("Susie")	TATAAAAA	TAGGTCTGTT	CAGGTCTGTTTT
7	*Pongo pygmaeus abelii*	Sumatran Orangutan	Coriell*	PR01109 ("Susie")	TATAAAAA	TAGGTCTGTT	CAGGTCTGTTTT
8	*Pongo pygmaeus abelii*	Sumatran orangutan	Coriell	GM06213A	TATAAAAA	TAGGTCTGTT	CAGGTCTGTTTT
9	*Pongo pygmaeus abelii*	Sumatran orangutan	Coriell	GM04272A	TATAAAAA	TAGGTCTGTT	CAGGTCTGTTTT
10	*Pongo pygmaeus abelii*	Sumatran orangutan	Coriell	NG12256	TATAAAAA	TAGGTCTGTT	CAGGTCTGTTTT
11	*Pongo pygmaeus abelii*	Sumatran orangutan	Coriell	NG06209	TATAAAAA	TAGGTCTGTT	CAGGTCTGTTTT
12	*Pongo pygmaeus pygmaeus*	Bornean Orangutan	Coriell	AG05252A	TATAAAAA	TAGGTCTGTT	CAGGTCTGTTTT
13	*Pongo pygmaeus pygmaeus*	Bornean Orangutan	SDFZ	KB4204	TATAAAAA	TAGGTCTGTT	CAGGTCTGTTTT
14	*Pongo pygmaeus pygmaeus*	Bornean Orangutan	SDFZ	KB5404	TATAAAAA	TAGGTCTGTT	CAGGTCTGTTTT
15	*Pongo pygmaeus pygmaeus*	Bornean Orangutan	SDFZ	KB5405	TATAAAAA	CAGGTCTGTT	CAGGTCTGTTTT

IPBIR: Integrated Primate Biomaterials and Information Resource
Coriell: Coriell Institute for Medical Research, 403 Haddon Avenue, Camden, NJ
SDFZ: San Diego Frozen Zoo,
Conservation and Research for Endangered Species (CRES)
* DNA PR01109 in conjunction with the Orangutan Genome Sequencing Project
Washington University Genome Sequencing Center, St. Louis, MO 63108
