# Supplementary material for: Orangutan Alu quiescence reveals possible source element: support for ancient backseat drivers
Source: Mob DNA. 2012 Apr 30;3:8. doi: 10.1186/1759-8753-3-8 (PMC3357318; doi:10.1186/1759-8753-3-8)
Supplement: Additional file 3 — Table S2. This file provides FASTA output for three of the potential secondary source elements in the orangutan from the [ponAbe2] genome assembly and Additional file 2: Table S2. [file 1759-8753-3-8-S3.PDF]

Additional File 3

Orangutan Chr 21: 23655135-23655823

```
>ponAbe2_dna range=chr21:23655135-23655823 5'pad=200 3'pad=200 strand=+
repeatMasking=lower
ATGAAAAAACCCTGCATAGTCATTAGGTGAGAGAAGAAATTCAGAACACC
ATGTCTTCACTCTGTATGTGTACATCCTGGAGATGAAGAATTATAGATTA
AAAAACCAAAATTACTATTTAATTATTTTGTATAACAAAATGAGACACTA
CCATCACTTTTTGAGACACTTACATGTATGTCAACCAAAAAGCAAAGCCC
ggccgggcgcggtggctcacgcctgtaatcccagcactttgggaggccga
ggcgggcggatcacgaggtcaggagatcgagatcatcctggctaacacgg
tgaaaccccgtctctactaaaaatacaaaaaattagcctggcgaggtggc
gggcgcctgtagtcccagctactcgggaggctgaggcaggagaatggcgt
gaacccagggggcggagcctgcaatgagctgagatcgcgccactgcactc
cagcctgggcgatagcgagcctccgtctcaaaaaaaaaaaaaaaaaaaaa
aaaaaaccaaaaaaaaaacaaaaaaGCAAAGCCCAATTTTTCTTCCCCATA
CAACAGACTATCACATAAACTCTGCCCTGTGAGGGCCCAGGCCATCTAAT
TACAGCATTGCAGCTATCACAACTAAGCCAAGTTTCTCAGGAGACAAAAA
GCCACCTGTTTCTAGCCACACAATTAAGCCATGTAAACA
```

SW score	perc div.	perc del.	perc ins.	query sequence	position in query begin	end	(left)		matching repeat repeat	class/family	position in repeat begin	end	(left)	ID
2778	2.3	0.3	0.0	ponAbe2_dna	201	506	(183)	+	AluYf4	SINE/Alu	1	307	(5)	1

Not in human or chimp

Orangutan Chr 2b: 3809176-3809864

```
>ponAbe2_dna range=chr2b:3809176-3809864 5'pad=200 3'pad=200 strand=+
repeatMasking=lower
atatattatatatcatatataatatattatatatacatatataatatata
catatataatatattatatatacatatataaataCTTtttttactttaga
agttttacatttatagaaaagtttccaaaagagcttatgagtatagcaca
gagtttgcccacactcctcacgcatttctcttgctattaacatcttatac
ggccgggcgcggtggctcacgcctgtaatcccagcactttgggaggccga
ggcgggcggatcacgaggtcaggagatcgagatcatcctggttaacacgg
tgaaaccccgtctctactaaaaacacaaaaaattagcctggcgaggtggc
gggcgcctgtagtcccagctactcgggaggctgaggcaggagaatggcgt
gaacccagggggcggagcctgcagtgagctgagatcgcgccactgcactc
cagcctgggcgatagcgagcctccgtctcaaaaaaaaaaaaaaaaaaaaa
aaaaaaatcttatactactatggtacatttgtcatgactaattcaccagt
attggtacatcattattagtaactcaactccatactttgttcacatttcc
ttagtttttccttaatgtcctttttctgtttcaagatcttatccaaatga
ccacattccatttagtggtcatatctccttattctcttt
```

SW score	perc div.	perc del.	perc ins.	query sequence	position in query begin	end	(left)		matching repeat repeat	class/family	position in repeat begin	end	(left)	ID
285	5.8	5.8	0.0	ponAbe2_dna	28	79	(610)	+	(TA)n	Simple_repeat	2	56	(0)	1
1030	20.1	2.0	8.0	ponAbe2_dna	80	200	(489)	C	**L1MC3**	**LINE/L1**	(7)	7778	7666	2
2770	2.6	0.3	0.0	ponAbe2_dna	201	507	(182)	+	**AluYf4**	**SINE/Alu**	1	308	(4)	3
1030	20.1	2.0	8.0	ponAbe2_dna	508	688	(1)	C	**L1MC3**	**LINE/L1**	(120)	7665	7493	2

Not in human or chimp

Orangutan Chr 17: 56932516-56933204

```
>ponAbe2_dna range=chr17:56932516-56933204 5'pad=200 3'pad=200 strand=+
repeatMasking=lower
CTGGTCCTTGCTTTGCTGTCTTTGGAGGCCTACTCCAGTTGCTTTTTTTT
TTTTCCCAAGCAAGTTTTTACAACAAGGAGATACCCAAAAGAAAATTCTC
CAAGACAGGAATGCTTCCAAATTGTATTCGGTACAAAATGCTGTTGAACG
GCTCTACCTTTAGAAGGACGCAGAAGCACTGGCATTAAAACCCCCTTTCG
ggccgggcgcggtggctcacgcctgtaatcccagcactttgggaggccga
ggcgggcggatcacgaggtcaggagatcgagatcatcctggctaacacgg
tgaaaccccgtctctactaaaaatacaaaaaattagcctggcgaggtggc
gggcgcctgtagtcccagctactcgggaggctgaggcaggagaatggcgt
gaacccagggggcggagcctgcagtgagctgagatcgcgccactgcactc
cagcctgggcgatagcgagcctccgtctcaaaaaaaaaaaaaaaaaaaaa
aaaaaaaaaCCCCTTTCGGTGCACGGGGAGGGCCTGGGGCCTTGGATCTA
CCCGAGGGTTTTACGCTCCTCCAGACCCTATCAACAGCCTCACTAGGAAC
GTGGACGGTGGCAGACAGGAGCCCTGCCCCTGGGGAGCCAAAAACTCCAC
CAGGCAATTGTCCCGGTCTGATGAGAAGGTGTAGACACA
```

SW score	perc div.	perc del.	perc ins.	query sequence	position in query begin	end	(left)		matching repeat repeat	class/family	position in repeat begin	end	(left)	ID
2820	1.9	0.3	0.0	ponAbe2_dna	201	509	(180)	+	AluYf4	SINE/Alu	1	310	(2)	1

Not in human or chimp

Table S2. Number of non-diagnostic random mutations versus allele frequency

CHR	START	END	Distribution	Freq.	CpG	non-CpG
12	90005006	90005295	Poly S/B	0.5405	1	0
13	109637257	109637546	Poly Sumatran-specific	0.2027	2	0
17	56932716	56933004	Poly Sumatran-specific	0.0811	0	0
21	23655335	23655623	Poly Sumatran-specific	0.0946	0	1
2b	3809376	3809664	Poly Sumatran-specific	0.3513	0	2
17	18571864	18572153	Orangutan specific	1.0000	6	1
4	170477976	170478265	Orangutan specific	1.0000	2	5
