# Supplementary material for: Orangutan Alu quiescence reveals possible source element: support for ancient backseat drivers
Source: Mob DNA. 2012 Apr 30;3:8. doi: 10.1186/1759-8753-3-8 (PMC3357318; doi:10.1186/1759-8753-3-8)
Supplement: Additional file 5 — This file provides the Alu subfamily consensus sequences for the three youngest orangutan-specific subfamilies. [file 1759-8753-3-8-S5.PDF]

Additional file 5

The three youngest orangutan-specific *Alu* subfamily consensus sequences

*Alu*Yc1a5_*Pongo*

GGCCGGGCGCGGTGGCTCAAGCCTGTAATCCCAGCACTTTGGGAGGCCGAGGCGGGCGGAT
CACGAGGTCAGGAGATCGAGACCACGGTGAAACCCCGTCTCTACTAAAAATACAAAAAATT
AGCCGGGTGCGGTGGCGGGCGCCTGTAGTCCCAGCTACTCAGGAGGCTGAGGCAGGAGAAT
GGCGTGAACCCGGGAGGCGGAGCTTGCAGTGAGCCGAGATTGAGCCACTGCACTCCAGCCT
GGGCGACAGAGCGAGACTCCGTCTCAAAAA

*Alu*Ye5a2_*Pongo*

GGCCGGGCGCGGTGGCTCACGCCTGTAATCCCAGCACTTTGGGAGGCCGAGGCGGGCGGAT
CACGAGGTCAGGAGATCGAGACCATCCTGGCTAACACGGTGAAACCCCGTCTCTACTAAAA
ATACAAAAAATTAGCCGGGCGAGGTGGCGGGCGCCTGTAGTCCCAGCTACTCGGGAGGCTG
AGGCAGGAGAATGGCGTGAACCCCGGGGGGCGGAGCCTGCAGTGAGCCGAGATCGCGCCA
CCGCACTCCAGCCTGGGCAACAGCGAGACTCCGTCTCAAAAA

*Alu*Ye5b5_*Pongo*

GGCCGGGCGCGGTGGCTCACGCCTGTAATCCCAGCACTTTGGGAGGCCGAGGCGGGCGGAT
CACGAGGTCAGGAGATCGAGACCATCCTGGCTAACACGGTGAAACCCCGTCTCTACTAAAA
ATACAAAAAATTAGCCTGGCGAGGTGGCGGGCGCCTGTAGTCCCAGCTACTCGGGAGGCTG
AGGCAGGAGAATGGCGTGAACCCCAGGGGGCGGAGCCTGCAGTGAGCTGAGATCGCGCCAC
TGCACTCCAGCCTGGGCGATAGCGAGCCTCCGTCTCAAAAA
